# Glucocorticoid Treatment in Community-Acquired Pneumonia

**DOI:** 10.1055/a-2704-6851

**Published:** 2025-10-14

**Authors:** Pierre-François Dequin, Marco Confalonieri

**Affiliations:** 1 Inserm UMR 1100 Centre d'Etudes des Pathologies Respiratoires, Université de Tours, Tours, France; 2 Service de Médecine Intensive, Réanimation et Centre d'Investigation Clinique INSERM CIC 1415, Hôpital Bretonneau, CHU de Tours, Tours, France; 3 CRICS-TriGGERSep F-CRIN Network; 4 Department of Medical, Surgical and Health Sciences, University of Trieste, Trieste, Italy; 5 Pulmonology Unit, University Hospital of Cattinara, Trieste, Italy

**Keywords:** corticosteroids, glucocorticoids, community-acquired pneumonia, COVID-19

## Abstract

Despite a fairly large number of comparative trials (which are, however, very heterogeneous), the role of corticosteroids in the adjuvant treatment of community-acquired pneumonia remains controversial. Nevertheless, recent randomized trials with adequate power in intensive care unit patients, albeit with conflicting results, have contributed to clarifying our understanding of this issue. More accurate phenotyping of patients likely to benefit from corticosteroid treatment must now be performed. In COVID-19 pneumonia, their benefit is not in question. For certain specific pathogens, including viral pathogens, their indications must be refined. They are still not recommended for influenza. They appear generally safe for short-term use in select populations.

Community-acquired pneumonia (CAP) was the seventh leading cause of death in the world in 2021, with an age-standardized rate of deaths of 28.7/100,000 (95% confidence interval [CI]: 26.0–31.1). 1 These figures do not consider mortality linked to the COVID-19 pandemic, a particular form of CAP, which was the second leading cause of death worldwide in the same year. The mode of contamination by airborne droplets of most respiratory infections explains their high prevalence. Apart from pandemics, large epidemiological studies estimate the 1-month mortality rate among patients hospitalized for CAP in high-income countries is approximately 10 to 12%, 2 3 and between 16 and 23% in low-income countries. 4 For patients admitted to intensive care units (ICUs) in high-income countries, it ranges from 25 to 30%. 5 6 CAP may cause severe pulmonary and systemic inflammation, impairing gas exchange and leading to sepsis, organ failure, ARDS, or septic shock. 7 8 Death may result in the inability to correct hypoxemia (which is closely linked to the more or less sophisticated means available), the decompensation of underlying disease, the onset of septic shock, 9 a delay in administering appropriate anti-infective treatment, or the decision to limit treatment. Glucocorticoids possess powerful anti-inflammatory and immunomodulatory activities that may counteract the dysregulated inflammatory response 10 and help prevent complications. However, clinical trials evaluating glucocorticoids in CAP have yielded mixed results. The objective of this review is to summarize the current body of evidence and identify key questions that remain to be addressed.

## Key Elements of Glucocorticoid Pharmacology

Natural corticosteroids are composed of two main hormones synthesized from cholesterol in the adrenal cortex: aldosterone, derived from the mineralocorticoid pathway, and cortisol, derived from the glucocorticoid pathway. Cortisol, known as hydrocortisone in therapeutic use, has both glucocorticoid and mineralocorticoid properties. Synthetic glucocorticoids are derived from cortisol and were developed to maximize glucocorticoid effects (notably anti-inflammatory and immunomodulatory) while minimizing mineralocorticoid effects. 11 12 Table 1 summarizes the glucocorticoid and mineralocorticoid potencies of various corticosteroids relative to hydrocortisone.

**Table 1:** Relative potencies and equivalent doses of different corticosteroids

	Equivalent dose (mg)	Glucocorticoid activity	Mineralocorticoid activity	Duration of action (h)	HPA axis suppression potency
Natural steroids					
Cortisol = hydrocortisone	200	1	1	8–12	1
Aldosterone	–	0.3	3,000		
Synthetic steroids					
Cortisone	250	0.8	0.8	8–12	0.8
Fludrocortisone	–	10	125		
Prednisone	50	4	0.8	12–36	4
Prednisolone	50	4	0.8	12–36	4
Methylprednisolone	40	5	0.5	12–36	4
Betamethasone	7.5	25	0	36–72	
Dexamethasone	7.5	25	0	36–72	17

Abbreviation: HPA, hypothalamic–pituitary–adrenal.

Equivalent doses are expressed for standard doses used in therapeutic trials. They apply only to oral and intravenous administration. Glucocorticoid activity reflects anti-inflammatory potency. Mineralocorticoid activity is often expressed as sodium retention power, but should not be considered merely as an undesirable side effect.

Corticosteroids have very good bioavailability, making them suitable for oral administration. When administered intravenously, they are in the form of hydrophilic esters that are converted into active forms within a few minutes. Their protein binding (albumin for all, and additionally transcortin for hydrocortisone and prednisolone) is moderate. Only unbound (free) cortisol can enter cells and bind to the glucocorticoid receptor (GRα). Their apparent distribution volume is moderate. Their metabolism is enzymatic and intracellular. Renal excretion in native form is very low. 11 13

Corticosteroids enter the cytoplasm by passive diffusion, where they bind to the α isomer of GRα, which plays a central role in their pharmacodynamic effects. 11 14 GRα is ubiquitously found in almost every cell type throughout the body, exhibiting pleiotropic effects of its activation, meaning it influences a wide range of biological processes, mostly tissue- and organ-specific. 15 Most of these effects are genomic, meaning that they occur primarily through messenger RNA transcription. They therefore appear relatively slowly (protein synthesis can begin within 30 minutes, but effects on functions and organs take several hours or days to appear) and also persist after the drug has been eliminated. 11 These transcriptional effects involve both the activation and repression of a large number of genes—approximately 20% of the human genome is sensitive to the action of corticosteroids. 16

Added to this are posttranscriptional effects (modification of mRNA stability), translational effects (modifications of ribosomes), and posttranslational effects (modifications of certain proteins).

Nongenomic effects are less well understood. They appear at higher plasma concentrations and develop more rapidly. They would involve interactions with other elements of the protein complex carrying GRα, with other receptors, or with the cell membrane. 11 14

The cellular and molecular effects of corticosteroids are therefore extremely numerous and varied, and their interactions are complex. 11 12 14 Today, the focus is on the dynamics of their effects, which include strengthening innate immunity, controlling inflammation, limiting tissue damage from acute aggression, and contributing to the development of acquired immunity and the restoration of cellular homeostasis. 14

## Controlled Trials of Corticosteroids in CAP

We found 18 published randomized trials comparing a corticosteroid with either a placebo or the standard of care. 17 18 19 20 21 22 23 24 25 26 27 28 29 30 31 32 33 34 Their main characteristics and results are summarized in Table 2 . Only seven involved more than two centers. 20 28 29 31 32 33 34 The number of patients included ranged from 30 19 to 795. 33 Seven trials included only or mostly intensive care patients. 20 21 26 29 32 33 34 Fourteen used a placebo as a comparator, including 11 in a double-blind design. Eight trials tested hydrocortisone, most often intravenously, 17 19 20 21 26 27 33 35 four prednisolone, 18 22 23 30 one prednisone, 28 three methylprednisolone 24 29 32 and two dexamethasone. 5 31 The duration of treatment ranged from a single injection 19 to 20 days. 32 The largest trials (more than 100 patients enrolled) were classic randomized superiority trials against placebo with a frequentist analysis, 23 25 28 29 30 31 32 33 with the exception of one in which hydrocortisone was compared with the standard of care, using a Bayesian approach. 34 Only three chose mortality as the primary outcome, all conducted in ICUs. 32 33 34 These studies, therefore, appear to be extremely heterogeneous.

**Table 2:** Comparative clinical trials of corticosteroids in CAP

Study; year	Country; no. of centers	Type of trial	Proportion of ICU patients at inclusion (%)	CTx; total dose; duration; no. of patients	Type of control; no. of patients	Principal outcome		Short-term mortality; RR; (CI 95%)	Notes
						CTx	Control		
						*p* -Value			
Wagner et al; 1956 17	The United States; 1	Open; pseudo-RCT	0	Oral HC; 560 mg; 5 d; *n* = 52	SoC; *n* = 33; or PLA; *n* = 28	Unclear	1.17 (0.07–18.89)		
McHardy and Schonell; 1972 18	Scotland; 1	Open RCT	0	Oral prednisolone; 140 mg; 7 d; *n* = 20	SoC; *n* = 20	Unclear	0.50 (0.05–5.62)		
Marik et al; 1993 19	South-Africa; 1	Open RCT	0	IV HC; 10 mg/kg; Single dose; *n* = 14	PLA; *n* = 16	TNF-α; blood level	NA	
						No significant change with time		
Confalonieri et al; 2005 20	Italy; 6	Double-blind RCT	100	IV HC; 1,880 mg; 7 d; *n* = 23	PLA; *n* = 23	PaO _2_ :FiO _2_ on D7			PaO _2_ :FiO _2_ : *p* = 0.0008; MODS: *p* = 0.03; Mortality on D60: 0/23 vs. 8/23, *p* = 0.01
						332 ± 80	237 ± 92		
						MODS on D7			
						0.3 ± 0.5	1.0 ± 0.9		
El-Ghamrawy et al; 2006 21	Saudi Arabia; 1	Double-blind RCT	100	IV HC; 1,880 mg; 7 d; *n* = 17	PLA; *n* = 17	Hospital mortality		0.50 (0.13–2.05)	
						3/17	6/17		
						NS			
Mikami et al; 2007 22	Japan; 1	Open RCT	0	IV prednisolone; 120 mg; 3 d; *n* = 15	SoC; *n* = 16	HLOS (d)		NA	Clinical stability earlier and ABx duration shorter in the CTx group
						11.3 ± 5.5	15.5 ± 10.7		
						*p* = 0.182			
Snijders et al; 2010 23	NL; 1	Double-blind RCT	0	Oral prednisolone; 280 mg; 7 d; *n* = 104	PLA; *n* = 104	Clinical cure at D8		1.05 (0.34–3.28)	Resolution of fever and CRP decreases faster in the CTx group
						80.0%	85.3%		
						OR: 0.72; 95% CI (0.35–1.49)			
Fernández-Serano et al; 2011 24	Spain; 1	Double-blind RCT	Unclear	MPD; 620 mg; 9 d; *n* = 23	PLA; *n* = 22	Mechanical ventilation		0.96 (0.06–15.51)	Clinical stability earlier, CRP and IL-6 decrease faster in the CTx group
						1/23	5/22		
						NS			
Meijvis et al; 2011 25	NL; 2	Double-blind RCT	0	DXM; 20 mg; 4 d; *n* = 151	PLA; *n* = 153	HLOS (d)		0.83 (0.34–2.01)	CRP and IL-6 decrease faster in the CTx group
						6.5 (5.0–9.0)	7.5 (5.3–11.5)		
						*p* = 0.048			
Sabry and Omar; 2011 26	Egypt; 2	Double-blind RCT	100	IV HC; 2,300 mg; 7 d; *n* = 40	PLA; *n* = 40	PaO _2_ :FiO _2_ on D7		0.33 (0.07–1.67)	PaO _2_ :FiO _2_ : *p* = 0.0008; MODS: *p* = 0.003
						338 (39)	243 (45)		
						MODS on D7			
						1.1 (0.53)	03.0 (0.9)		
Nafae et al; 2013 27	Egypt; 1	Single-blind RCT	Unclear	IV HC; 2,300 mg; 7 d; *n* = 60	PLA; *n* = 20		0.23 (0.06–0.82)		
Blum et al; 2015 28	Switzerland; 7	Double-blind RCT	Unclear (low)	Oral prednisone; 140 mg; 7 d; *n* = 392	PLA; *n* = 393	Time to clinical stability (d)		1.23 (0.59–2.57)	More adverse effects and shorter HLOS; In the CTx group
						3.0 (2.5–3.4)	4.4 (4.0–5.0)		
						*p* < 0.0001			
Torres et al; 2015 29	Spain; 3	Double-blind RCT	75	IV MPD; 5 mg/kg; 5 d; *n* = 61	PLA; *n* = 59	Treatment failure (composite outcome)		0.64 (0.23–1.83)	Fewer late treatment failures (72–120 h) based on radiological progression criteria
						8/61	18/59		
						*p* = 0.02			
Lloyd et al; IMPROVE-GAP; 2019 30	Australia; 2	CTx domain of a four-bundle stepped-wedge cluster open RCT	0	Oral prednisolone; 350 mg; 7 d; *n* = 105	PLA; *n* = 292	HLOS (d)		0.96 (0.49–1.93)	
						3.0 (1.9–4.6)	3.0 (1.9–4.7)		
						NS			
Wittermans et al; SANTEON-CAP; 2021 31	NL; 4	Double-blind RCT	0	Oral DXM; 24 mg; 4 d; *n* = 203	PLA; *n* = 198	HLOS (d)		0.56 (0.16–1.91)	Stopped early for slow recruitment. Admission to ICU 5/203 vs. 14/198 *p* = 0.03
						4.5 (4.0–5.0)	5.0 (4.6–5.4)		
						*p* = 0.033			
Meduri et al; ESCAPe; 2022 32	The United States; 42	Double-blind RCT	100	IV MPD; 508 mg; 20 d; *n* = 297	PLA; *n* = 287	Mortality on D60		0.91 (0.61–1.36)	Stopped early for slow recruitment and futility
						47/286 (16.4%)	50/277 (18.1%)		
						*p* = 0.61			
Dequin et al; CAPE COD; 2023 33	France; 31	Double-blind RCT	100	IV HC; 1,100–1,950 mg; 8–14 d; *n* = 400	PLA; *n* = 395	Mortality on D28		0.53 (0.32–0.86)	Stopped early after the second interim analysis
						25/400 (6.2%)	47/395 (11.9%)		
						*p* = 0.006			
Angus; REMAP-CAP; 2025 34	International (18 countries); 101	CTx domain of an open randomized adaptive trial	100	IV HC; 1,400 mg; 7 d; *n* = 536	SoC; *n* = 122	Mortality on D90		1.52 (0.81–2.80)	Stopped for futility; Very low probability of benefit (Bayesian analysis)
						78/521 (15.0%)	12/122 (9.8%)		
						NS			

Abbreviations: CI, confidence interval; CTx, corticosteroid; DXM, dexamethasone; HC, hydrocortisone; HLOS, hospital length-of-stay; MODS, multiple organ dysfunction score; MPD, methylprednisolone; NL, the Netherlands; PLA, placebo; RR, relative risk; SoC, standard-of-care.

We found 31 meta-analyses published in English in this field, often without taking this clinical heterogeneity into account. They classified some of the above studies as having a high or nonassessable risk of bias, without always excluding them from the analysis. Furthermore, the publication of each recent trial 32 33 34 was quickly followed by a new meta-analysis, 35 36 37 often with different criteria for selecting the trials included, making comparisons difficult. We will therefore focus the rest of this section on analyzing the main trials, raising hypotheses about the discrepancy in their results.

### Trials Conducted in the Ward

Five large trials evaluated adjuvant corticosteroid therapy in noncritically ill patients. One study compared 40 mg of prednisolone per day for 7 days with a placebo in 208 patients and found no significant difference in the proportion of patients who were clinically cured on day 8. Resolution of fever and decrease in C-reactive protein (CRP) level were faster when patients received corticosteroids. 23 In contrast, the Swiss STEP trial showed that with the same daily and total dose of prednisone, the median time to clinical stability (based on predefined criteria) was 3 days in the 392 patients who received the corticosteroid and 4.4 days in the 393 who received placebo, a significant difference. 28 As a secondary outcome, the length of hospital stay was also reduced with prednisone. These results were not influenced by the documented pathogens (or the fact that none were documented), the antibiotic therapy administered, the procalcitonin level, or the absence of fever at inclusion, given the limitations of multiple comparisons and the small size of some subgroups. 38 Two trials conducted in the Netherlands showed that dexamethasone administered for 4 days at a dose of 5 or 6 mg/day, respectively, reduced the median length of hospital stay by 1 day in the first trial and by half a day in the second trial. 5 31 In the latter, the proportion of patients who were subsequently transferred to the ICU was lower among those receiving corticosteroids. 31 Finally, in an Australian four-bundle stepped-wedge cluster open trial involving a corticosteroid domain, prednisolone failed to reduce the hospital stay. 30 None of these trials with low mortality rates showed any benefit in terms of survival.

In summary, trials with the lowest risk of bias conducted outside ICUs show consistent evidence that synthetic corticosteroids administered for 4 to 7 days accelerate recovery and slightly reduce the length of hospital stay, albeit at the cost of slightly more frequent side effects, mainly hyperglycemia. 28

### Trials Conducted in the ICU

Similarly, among the trials that evaluated corticosteroids in intensive care patients, five will be detailed. Published 20 years ago, one of them was a cornerstone in this field, as it was the first to revive interest in this issue. It randomized a relatively small number of patients (23 in each group) to receive either hydrocortisone 10 mg/hour for 7 days or a placebo. This trial showed that on day 7, the PaO _2_ :FiO _2_ ratio and organ failure score were significantly higher and lower, respectively, in patients receiving corticosteroids. As a secondary outcome, no patients treated with hydrocortisone died by day 60, compared with eight of the patients who received a placebo. 20

Ten years later, a study conducted in Barcelona was published, with the unique feature of including only patients (75% of whom were hospitalized in the ICU) with a marked inflammatory syndrome with CRP greater than 15 mg/dL. Patients receiving 1 mg/kg/day of methylprednisolone for 5 days had significantly fewer treatment failures than those receiving a placebo. Treatment failure was defined by a composite endpoint, and the difference was based on radiographic progression between 72 and 120 hours after the start of treatment. 29

More recently, three large-scale trials have sought to demonstrate improved survival with corticosteroids, with conflicting results.

The ESCAPe trial 32 was designed to enroll 1,406 patients admitted to the ICU for CAP, with severity criteria from the American Thoracic Society/Infectious Diseases Society of America. 39 Randomization had to take place no later than 96 hours after admission to the hospital. The trial was stopped early due to slow recruitment after 584 eligible patients were enrolled (including 201 with healthcare-associated pneumonia), of whom 297 received methylprednisolone at an initial dose of 40 mg/day, gradually reduced from day 8, for a total treatment duration of 20 days. A placebo was administered to 287 patients. Forty-two centers from the Department of Veterans Affairs participated. The primary outcome was mortality at day 60. It was 16% in patients who received corticosteroids and 18% in those who received a placebo, a nonsignificant difference. No significant differences were observed for any of the numerous secondary outcomes.

The CAPE COD trial 33 had a relatively similar design: it was planned to randomize 1,200 patients to receive either hydrocortisone at a dose of 200 mg/day, gradually reduced from day 4 for patients whose condition had improved according to predefined criteria, or from day 8 for the others, with a maximum treatment duration of 8 and 14 days, respectively, or an industrially manufactured placebo. Treatment was discontinued upon discharge from the ICU, regardless of its duration. Patients were included within 24 hours of the onset of a severity criterion (mechanical ventilation or PaO _2_ :FiO _2_ less than 300 under high-flow nasal cannula or nonrebreathing mask oxygen therapy). Recruitment involved 31 French centers and had to be halted at the start of the COVID-19 pandemic after 800 patients were enrolled, of whom 795 were analyzable. After a planned interim analysis, the Data Safety and Monitoring Board recommended stopping enrollment, although the efficacy stop conditions (significant reduction in mortality at day 28) were not met at the defined threshold ( *p* < 0.001), due to the ongoing pandemic making continuation of the trial uncertain. The mortality rate at day 28 was 6.2% in patients treated with hydrocortisone and 11.9% in those receiving placebo, a significant difference ( *p* = 0.006). Among secondary outcomes, mortality was also reduced at day 90 (9.3 vs. 14.7%, *p* = 0.02), and the proportions of patients requiring tracheal intubation or vasopressor therapy after enrollment were significantly lower among those receiving hydrocortisone.

How can these contradictory results be explained? The populations studied appear to be fairly similar, with a median age of 69 years in the ESCAPe study 32 and 67 years in the CAPE COD study, 33 the same proportion of patients classified as class IV or V according to the lung injury score 40 (82.0 vs. 82.6%), and fairly similar proportions of patients treated with vasopressors (13.0 vs. 11.6%) or, to a lesser extent, with mechanical ventilation (33.0 vs. 44.4%, including noninvasive ventilation). However, there were notable differences between the two trials: the proportions of women included were different (3.8 vs. 30.6%), the median Sequential Organ Failure Assessment (SOFA) score seemed higher in ESCAPe (6.5 vs. 4), treatment was started earlier in CAPE COD and was shorter (median duration of 5 days), and only CAP (not healthcare-associated) was included. At the doses used, methylprednisolone and hydrocortisone are considered to have glucocorticoid effects of equal intensity. However, the mineralocorticoid effect is twice as strong with hydrocortisone ( Table 1 ). A heterogeneity in the treatment effect was suspected, and could be partly related to the inflammatory profile of the patients (see below). CRP was greater than 15 mg/dL in 69% of CAPE COD patients (23.3% missing data). These data have not been published for ESCAPe.

The third trial was the fixed-duration corticosteroids domain of the REMAP-CAP adaptive platform trial, in which 536 patients were randomized to receive 200 mg/day of hydrocortisone for 7 days, without tapering, and compared in an open-label fashion with 122 patients receiving standard of care. 34 Of the 101 centers in 18 participating countries, only 70 offered the option of including both arms, so the analyses also included a subgroup of 339 patients treated with hydrocortisone compared with 116 controls, all admitted to the centers recruiting in both arms. The recruitment was halted due to futility. Survival at day 90 was not significantly different between the two groups (85 vs. 90.2%), although there was a relative risk of death associated with hydrocortisone (relative risk [RR]: 1.52; 95% CI: 0.81–2.80). In a Bayesian analysis, the probability of superiority of hydrocortisone ranged from 9.2 to 15.7%, depending on the group. Conversely, the probability of harm ranged from 84.3 to 90.8%. Among secondary outcomes, the median number of organ support-free days was 24 days (confidence interval [IQR]: 16–26) in patients receiving hydrocortisone and 22.5 (IQR: 15–26) in controls, due to a shorter duration of cardio-vascular support, resulting in an 85.4% probability that hydrocortisone was superior to control on this endpoint. Overall, this trial concluded that adding a 7-day course of hydrocortisone to usual care was unlikely to improve survival, 34 contrasting with the results of previous trials. 20 33

Once again, what hypotheses can be made to explain these discrepancies? REMAP-CAP patients were slightly younger than CAPE COD patients (approximately 60 years old vs. 67 years old), were more often on invasive ventilation (39.0 vs. 22.3%), but with slightly higher median PaO _2_ :FiO _2_ ratios (156 in patients receiving hydrocortisone and 168 in controls, vs. 143 and 137, respectively). A higher proportion were treated with vasopressors (48.8 vs. 11.6%), without specifying the proportion of patients in septic shock at inclusion (a noninclusion criterion for the CAPE COD trial). Nevertheless, median lactate levels and their interquartile ratios were very similar between the two trials. The 90-day mortality rate in the control group was 9.8% in REMAP-CAP and 14.7% in CAPE COD, much lower than expected in patients admitted to the ICU, and not consistent with a high proportion of patients in septic shock.

Although the proportion of patients treated with vasopressors was very similar in both arms of REMAP-CAP (49% in patients receiving hydrocortisone and 47% in the control group), the cardiovascular SOFA score seems different: 2 (IQR: 0–3) versus 1 (0–3), and the difference was even more pronounced in the subgroup of patients included in centers offering a control group: 3 (IQR: 0–3) versus 1 (0–3), suggesting a possible imbalance in vasopressor doses between the two groups. REMAP-CAP was an open-label trial, which contributed to 23% of patients in the control group receiving a corticosteroid for a median duration of 4 days—a protocol violation that could not reverse the result. The standard of care in the control group was defined as that applied in each of the 101 centers in 18 countries, suggesting possible heterogeneity, potentially influenced by knowledge of the randomization group.

In summary, trials conducted in ICUs are inconsistent, perhaps partly due to differences in case mix or their inherent limitations, but they encourage further research to better characterize each patient's sensitivity to corticosteroids.

To date, only one meta-analysis has integrated the results of the three most recent trials, while also including trials that included sepsis and septic shock or acute respiratory distress syndrome, provided that the mortality rate in the CAP subgroup was known. 37 It concluded with moderate certainty that corticosteroids probably reduced 1-month mortality in CAP (RR: 0.82; 95% CI: 0.74–0.91), and with high certainty that they reduced the need for invasive mechanical ventilation (RR: 0.63; 95% CI: 0.48–0.82).

A meta-analysis of individual data from eight trials (not including the corticosteroid domain of REMAP CAP) concluded that there was a reduction in mortality at day 30 in patients receiving corticosteroids (odds ratio [OR]: 0.72; 95% CI: 0.56–0.94), but only in patients with a CRP greater than or equal to 20.4 mg/dL at inclusion. 41

### Role of Pathogens

With the exception of the oldest trial, which only included pneumococcal pneumonia, 17 all of the studies reviewed above were included on the basis of clinical and radiological criteria. As a result, the nature of the pathogens isolated depended on the investigations performed. No trials used systematic multiplex PCR screening. The isolated pathogens are therefore primarily bacteria, and documented viral pneumonia is rare in all these studies, contrasting with recent epidemiological findings that highlight the high proportion of viral pneumonia in patients admitted for CAP in U.S. hospitals. 42 However, in trials involving corticosteroids, among patients admitted to the ICU, the proportion of patients without an isolated pathogen ranged from 35 20 to 57%. 32 Among those treated in the ward, the percentage ranged from 37 18 to 76%. 28 It is possible that among these patients without documented infection, some had CAP diagnosed on the basis of clinical and radiological criteria but did not have true bronchopulmonary infection. However, it is also highly probable that many had a genuine infection, without the pathogen(s) having been isolated. The now established frequency of viral pneumonia and the absence in these studies of systematic testing for viral pathogens suggest that a significant proportion of patients had viral pneumonia.

Certain viruses that have been the subject of specific studies will be discussed below.

### Patients with Specific Profiles

#### Immunodeficiency

Severe immunodeficiency is most often a noninclusion criterion, meaning that the proportion of immunocompromised patients in trials is too small to assess the effect of corticosteroids in this population. A study of 7,449 patients hospitalized for CAP showed that 10% were immunocompromised, mainly due to advanced cancer, cancer chemotherapy, or prolonged corticosteroid therapy. 43 A pathogen was identified in 24% of patients, with a similar distribution of germs between immunocompetent and immunocompromised patients. However, very few cases of HIV infection, solid organ transplantation, or stem cell transplantation were reported. More than the pathogen itself, it is the host–pathogen interaction that will be modified by immunosuppression, with a poorly understood impact of corticosteroids. For safety reasons, it has therefore been suggested not to treat immunocompromised individuals with corticosteroids in cases of CAP 44 (see below for the specific case of pneumocystosis).

#### Septic Shock

Pneumonia is the most common cause of sepsis. 45 In a prospective cohort study of 4,070 patients hospitalized for CAP, 37.6% met the criteria for “severe sepsis” (as defined at the time). 46 The proportion of patients in septic shock at study entry in trials evaluating corticosteroids in CAP was 6%, 20 23%, 29 or not specified. Nevertheless, the first RCT showed a delayed septic shock rate by day 8 of 43% in the placebo arm, but not among the hydrocortisone-treated patients. 20 The proportion of patients receiving vasopressors at inclusion was 11.6% in the CAPE COD trial (where septic shock was a criterion for noninclusion, but where vasopressors were allowed in cases of hypotension secondary to sedation and positive pressure mechanical ventilation, with a maximum dose of norepinephrine of 0.25 μg/kg/minute). 33 This proportion was 13.0% in the ESCAPe trial (where the maximum dose of norepinephrine was set at 0.30 μg/kg/minute) 32 and 48.8% in the REMAP-CAP corticoids domain, 34 but with lactate levels and mortality rates that did not suggest a high proportion of septic shock (see discussion above).

On the other hand, corticosteroids, primarily hydrocortisone, have been extensively evaluated in sepsis and septic shock, with mixed results. 47 A recent meta-analysis of individual patient data concluded that hydrocortisone reduced the duration of vasopressor treatment in septic shock, and that only the combination of hydrocortisone and 9-α fludrocortisone (a potent synthetic mineralocorticoid) improved survival (death marginal RR: 0.86; 95% CI: 0.79–0.92). 48 Hydrocortisone alone did not improve survival, regardless of whether septic shock was related to a respiratory infection. The APROCCHSS trial showed a reduction in 90-day mortality in patients with severe septic shock (SOFA score: 12 ± 3), linked in 45.3% of cases to CAP, when they received a combination of hydrocortisone and 9-α fludrocortisone. 49 A post hoc analysis of this trial recently showed that improved survival was observed only in the subgroup of patients whose septic shock was secondary to CAP. 50 Overall, complicated CAP with septic shock is probably an excellent indication for corticosteroid treatment, with hydrocortisone and 9-α fludrocortisone combination therapy having the highest level of evidence.

#### Aspiration Pneumonia

No trials have specifically evaluated corticosteroids in inhalation pneumonia with a clinical outcome. Nevertheless, the ESCAPe trial recruited 34% of participants who met healthcare-acquired pneumonia criteria, which can be mostly caused by aspiration. 32 Thus, in the absence of data, they are not indicated. 44

#### Local Complications of Pneumonia

No data are available for pulmonary abscess. In the STOPPE pilot trial conducted in 79 patients with CAP complicated by pleural effusion, dexamethasone 8 mg/day for 48 hours showed no benefit over placebo, particularly with regard to the progression of pleurisy. 51

### Safety of Corticosteroids

The way in which each trial investigates and reports adverse effects varies greatly. Furthermore, no trial has the power to detect an increase in the risk of a given complication when it is rare in the control group. Meta-analyses are relevant here, even if they cannot compensate for the lack of systematic evaluations on certain complications.

The safety of corticosteroids can be summarized as follows: first, the risk of hyperglycemia is increased by corticosteroids, with a relative risk of 1.32 (95% CI: 1.12–1.56) in the most recent and comprehensive meta-analysis. 37 In the ICU, where hyperglycemia is common, particularly in septic patients, this may result in higher insulin doses being administered to patients than in the control group. 33 After 7 days of corticosteroid therapy in non-ICU patients, the frequency of insulin treatment at 1 month is no higher than when patients received a placebo. 28 Second, the frequency of hospital-acquired infections is no higher when corticosteroids are administered for CAP (RR: 0.97; 95% CI: 0.85–1.11), 37 nor is it higher for septic shock (RR: 1.04; 95% CI: 0.95–1.15). 48 However, patients of the STEP trial demonstrated a higher risk of recurrent pneumonia when they received prednisolone (OR: 2.57; 95% CI: 1.29–5.12). 28 Third, the occurrence of gastrointestinal bleeding is very rare and does not appear to be higher when corticosteroids are administered (RR: 0.86; 95% CI: 0.58–1.29). 37 Fourth, neuropsychiatric and neuromuscular complications should be investigated more systematically. Fifth, no adverse effects related to hypothalamic–pituitary–adrenal axis suppression were reported in clinical trials on patients with pneumonia receiving low-dose corticosteroid treatment for less than 2 or 3 weeks. However, the usual absence of biological tests of the axis and its reactivity limits the reliability of this data. A tapering strategy may be considered after 2 weeks of treatment. 14 Overall, severe side effects do not appear to be more common with corticosteroids (RR: 0.75; 95% CI: 0.57–0.99), 37 given that so-called “low doses” are used, usually for a short period of time, around 1 week. However, in the two largest trials conducted outside the ICU, secondary events (regardless of severity) were overall more frequent in the group receiving corticosteroids, and were dominated by hyperglycemia. 28 31

## Controlled Trials of Corticosteroids in the Treatment of Pneumonia Caused by Specific Pathogens

This section will briefly discuss the effects of corticosteroids in a few specific infections.

### Influenzae

The evaluation of corticosteroids in influenza is based solely on case-control or cohort studies, most often conducted in an epidemic setting. A meta-analysis of 21 of these studies conducted by the Cochrane group showed higher 1-month mortality in patients who received corticosteroids (OR: 3.90; 95% CI: 2.31–6.60). 52 Another meta-analysis of 15 studies dedicated to severe influenza-CAP (or complicated with ARDS) found a similar result (OR: 2.30; 95% CI: 1.68–3.16). However, excess mortality was no longer significant when only the five studies reporting adjusted mortality rates were included (adjusted OR: 1.31; 95% CI: 0.95–1.80). 53 A retrospective analysis of 1,846 ICU patients with severe influenza in Spain (32.7% treated with corticosteroids, mainly methylprednisolone) found an association between corticosteroid use and increased mortality (hazards ratio: 1.36; 95% CI: 1.08–1.60) using a propensity score–adjusted Cox model. 54 There have also been reports of an increase in the incidence of hospital-acquired infections with corticosteroid use in influenza, 53 with particular emphasis on the risk of aspergillosis. 55 In an animal model, exposure to corticosteroids prior to exposure to the influenza virus was associated with increased viral replication and suppression of genes involved in the innate antiviral immune response. 56 For all these reasons, corticosteroid therapy is not currently recommended for influenza, unless indicated for other reasons. There is an urgent need for high-quality randomized trials in this indication.

### SARS-CoV-2

Apart from supportive care, the first treatment to show benefit in severe forms of SARS-CoV-2 pneumonia was corticosteroid therapy. In a large, multicenter, adaptive platform trial in the UK, which included 6,425 patients, dexamethasone 6 mg/day showed a reduction in mortality at day 28 compared with standard treatment. 57 This benefit appeared to be influenced by the degree of respiratory failure: it was higher in patients on mechanical ventilation (29 vs. 41%; RR: 0.64; 95% CI: 0.51–0.81), intermediate in those receiving oxygen therapy, and nonexistent in patients breathing ambient air.

After the prepublication of these results, in the specific context of the first wave of the pandemic, other trials testing corticosteroid therapy were prematurely discontinued and often lacked statistical power. Nevertheless, a Brazilian multicenter trial concluded that dexamethasone increased the number of days without mechanical ventilation from 4.0 days (95% CI: 2.9–5.4) in the standard-of-care arm to 6.6 days (95% CI: 5.0–8.2), a significant difference. 58 Two trials tested hydrocortisone during this first wave. The hydrocortisone domain, at a fixed dose or adjusted to the duration of the shock, was opened in the REMAP-CAP adaptive platform trial. The outcome was organ support-free days. The Bayesian probability of superiority in favor of hydrocortisone was 93% for the fixed dose and 80% for the dose adjusted to the duration of shock. 59 Only one double-blind placebo-controlled trial was conducted, using the same design as the CAPE COD trial. The primary outcome was treatment failure at day 21, defined as death or dependence on mechanical ventilation or high-flow nasal cannula. Failure was observed in 42.1% of patients receiving hydrocortisone and 50.7% of those receiving placebo, a nonsignificant difference. 60 In post hoc analysis, the proportion of deaths at day 21 was 14.7 and 27.4%, respectively, a nonsignificant difference ( *p* = 0.06), but close to that observed later in the parent trial from which this study was derived. 33

Several trials have tested the hypothesis that a higher dose of dexamethasone is more effective. Two trials compared 6 and 12 mg/day in intensive care patients and found no significant benefit from the higher dose. 61 62 A trial by the RECOVERY group randomized adults to receive either standard care (including dexamethasone 6 mg/day in 87% of cases) or the same corticosteroid 20 mg/day for 5 days, followed by 10 mg/day for 5 days or until hospital discharge. An interim analysis showed excess mortality in the high-dose arm among patients receiving low-flow oxygen or breathing room air (19 vs. 12%; RR: 1.59; 95% CI: 1.2–2.1). Therefore, the trial was stopped in nonsevere patients. 63 In the meantime, a meta-analysis of eight trials (of which four at low-risk of bias) suggested little or no difference in mortality on day 30 when comparing 6 mg/day and more than 6 mg/day of dexamethasone in hypoxemic patients with COVID-19. 64 Similarly, a large RCT comparing higher dose methylprednisolone with conventional 6 mg/day dexamethasone did not find a mortality difference at day 28 in severe COVID-19 pneumonia. 65

### Other Coronaviruses: SARS and MERS

There are no randomized controlled trials evaluating corticosteroids in patients with severe acute respiratory syndrome or Middle Eastern respiratory syndrome. The few observational studies available do not allow conclusions to be drawn, and, to date, corticosteroids are not recommended in these situations, except in clinical research.

### Pneumocystis Jiroveci

In 251 patients with HIV infection and confirmed pneumocystosis, with varying degrees of hypoxemia but breathing spontaneously, prednisone (80 mg/day for 5 days, then 40 mg/day for 5 days, then 20 mg/day until the end of anti-infective treatment, or equivalent dose of methylprednisolone in patients requiring intravenous treatment) was associated on day 21 with a lower risk of respiratory failure (13 vs. 28%, *p* = 0.04) and death (9 vs. 18%, *p* = 0.024) than standard of care. The decrease in mortality observed with prednisone was greater in patients with more severe hypoxemia. 66 A meta-analysis by the Cochrane group, which included six trials and 489 patients, confirmed this result, with a 1-month mortality rate of 13% in patients who received corticosteroids and 25% in the control group (RR: 0.56; 95% CI: 0.32–0.98). 67

In patients with pneumocystosis not related to HIV infection, a meta-analysis of 16 observational studies involving 2,518 patients suggested higher mortality in patients receiving corticosteroids (OR: 1.37; 95% CI: 1.07–1.75), but with the opposite effect in patients defined as having acute respiratory failure, that is, with a partial oxygen pressure below 60 mm Hg in ambient air (OR: 0.63; 95% CI: 0.41–0.95). 68 In a recently published French multicenter trial, 226 patients with acute respiratory failure were randomized to receive either a placebo or methylprednisolone 30 mg twice daily for 5 days, then 30 mg daily for 5 days, then 20 mg daily until day 21. Mortality on day 8 was 32.4% in patients receiving placebo and 21.5% in those receiving corticosteroids, a nonsignificant difference ( *p* = 0.069). Among the secondary outcomes, hospital mortality and mortality at day 90 were significantly lower with corticosteroids, as was the proportion of patients requiring intubation after inclusion. 69

## Available Guidelines

The guidelines reflect the state of the literature at a given point in time. Thus, the U.S. recommendations, published in 2019, do not recommend the use of corticosteroids in CAP. 39 European and South American guidelines, written before the CAPE COD trial was published, only recommend corticosteroids in cases of septic shock complicating CAP. Strangely, they then suggest methylprednisolone, which has not been evaluated in septic shock. 70 More recently, a panel of international experts convened by the Society for Critical Care Medicine issued a strong recommendation, with moderate evidence, in favor of the use of corticosteroids in severe bacterial CAP in adults. The panel made no recommendation in the less severe CAP for adults, nor any recommendation for children. 71 The questionable reasons for restricting corticosteroids to bacterial pneumonia only have been discussed above. Furthermore, the REMAP CAP corticosteroids domain could not be considered in these recommendations. For COVID-19, the latest update to the WHO guidelines still strongly recommends corticosteroids, possibly combined with IL-6 receptor antagonists and baricitinib, for severe to critical cases. 72

## Outstanding Issues

It is highly unlikely that corticosteroids are a miracle cure suitable for all patients with CAP. It will be necessary to personalize their use according to clinical and biological phenotyping. A CRP threshold value suggested by a recent meta-analysis 41 supports this approach.

The analysis of biobanks created during recent large-scale trials could help to better define who can benefit from corticosteroids and in whom they may be harmful. This approach may also help to explain the seemingly conflicting results of certain trials.

Recently, the discovery of multiple isoforms of the GRα present in most tissues opened new questions on the need to better understand the contribution of the receptor to glucocorticoid sensitivity/resistance in clinical practice. 73

It will also be necessary to better define their place in populations that are often excluded from trials, particularly immunocompromised individuals, but also children.

The potential role of corticosteroids in severe influenza will need to be clarified through randomized trials with low risk of bias.

The effectiveness or otherwise of corticosteroids in partially preventing long-term complications of CAP will require trials with prolonged follow-up. Patients and their families should be involved in defining relevant outcome criteria.

The choice of medication used and how it is administered are likely to be important, and in this sense, one should be wary of believing in a “class effect.” To date, only hydrocortisone administered intravenously for a few days has shown a survival benefit, 20 27 33 although an open-label study concluded recently that the likelihood of benefit was very low. 34 The role of mineralocorticoid receptor stimulation deserves further investigation, which is not incompatible with the anti-inflammatory effect generally considered to be the mechanism of action of corticosteroids in CAP.
